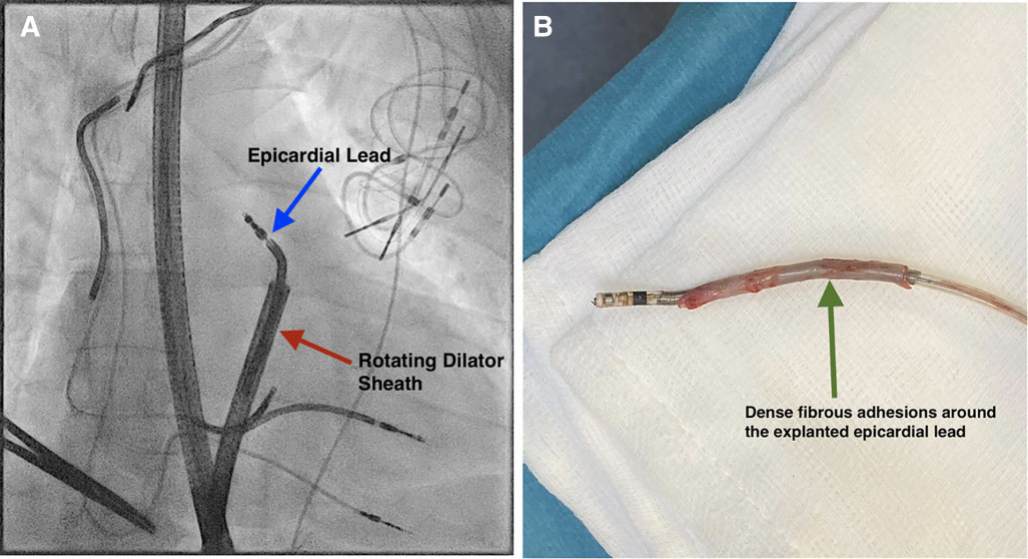# First use of a rotating mechanical dilator sheath to extract an epicardial defibrillator lead from the pericardial space

**DOI:** 10.1093/europace/euac060

**Published:** 2022-08-04

**Authors:** Vishal S Mehta, Mark K Elliott, William Regan, Jonathan M Behar, Eric Rosenthal, Christopher A Rinaldi

**Affiliations:** Department of Cardiology, Guy’s and St Thomas’ NHS Foundation Trust, London, UK; School of Biomedical Engineering and Imaging Sciences, St Thomas’ Hospital, King’s College London, London SE1 7EH, UK; Department of Cardiology, Guy’s and St Thomas’ NHS Foundation Trust, London, UK; School of Biomedical Engineering and Imaging Sciences, St Thomas’ Hospital, King’s College London, London SE1 7EH, UK; Department of Paediatric Cardiology, Evelina London Children’s Hospital, London, UK; Department of Cardiology, Guy’s and St Thomas’ NHS Foundation Trust, London, UK; Department of Paediatric Cardiology, Evelina London Children’s Hospital, London, UK; Department of Cardiology, Guy’s and St Thomas’ NHS Foundation Trust, London, UK; School of Biomedical Engineering and Imaging Sciences, St Thomas’ Hospital, King’s College London, London SE1 7EH, UK

A 39-year-old male with an implantable cardioverter defibrillator was referred for device extraction. Multiple system failures resulted in the implant of an epicardial system with a single coil (Sprint Quatro, Medtronic, MN, USA) placed in the posterior pericardium, a single coil (Transvene, Medtronic, MN, USA) placed subcutaneously, and a Capsurefix 5076 (Medtronic, MN, USA) pace-sense lead all tunnelled to a subrectus generator.

Two years later, positron emission tomography–computed tomography confirmed system infection. Explant of the infected material occurred in a hybrid theatre. A subxiphoid incision was performed for direct visualization of the pericardial space. The proximal portion of the pericardial lead was visualized using a Convergent introducer sheath (Atricure, West Chester, OH, USA) and thoracoscope; however, the tip was not visualized. The lead was prepared with retraction of the screw mechanism and a lead locking device (LLD EZ™, Philips Healthcare, USA) stylet was deployed. Gentle manual traction was applied, but the lead was adhered within the pericardial space. A rotating dilator sheath (13F 545-513 Tightrail, Spectranetics, CO, USA) under direct visualization was used to successfully extract the pericardial lead (*Panel A*). The outer sheath was not used as venous access did not need to be maintained and allowed greater flexibility of the tool. Mechanical extraction was undertaken to free the lead from the fibrous binding sites (*Panel B*).

We describe the first epicardial shock lead extracted with a rotating mechanical cutting tool aided by direct visualization, thereby avoiding sternotomy.

The full-length version of this report can be viewed at: https://www.escardio.org/Education/E-Learning/Clinical-cases/Electrophysiology.

**Figure:**